# Arteannuin B Induces Ferroptosis in Colorectal Cancer Cells via GDF15/HMGCS1/GPX4 Axis

**DOI:** 10.7150/jca.128611

**Published:** 2026-07-20

**Authors:** Xiaozheng Ou, Jinghong Xu, Yuting Jin, Yanqing Wang, Rongmin Yu, Jianhua Zhu, Weijuan Huang, Liyan Song

**Affiliations:** 1 Biotechnological Institute of Chinese Materia Medica, Jinan University, Guangzhou, China.; 2 Department of Pharmacology, College of Pharmacy, Jinan University, Guangzhou, China.; 3 Department of Natural Medicinal Chemistry, College of Pharmacy, Jinan University, Guangzhou, China.

**Keywords:** Arteannuin B, Colorectal cancer, Ferroptosis, Mevalonate pathway, Growth differentiation factor 15.

## Abstract

**Purpose:**

Drug resistance in colorectal cancer (CRC) necessitates novel therapeutic strategies. This study investigated whether arteannuin B, a sesquiterpene lactone from *Artemisia annua*, induces ferroptosis in CRC cells and elucidated the underlying molecular mechanism.

**Methods:**

Anti-CRC effects were assessed *via* MTT assays and xenograft models. Proteomics identified differentially expressed proteins. Arteannuin B-induced ferroptosis was confirmed by measuring reactive oxygen species (ROS), lipid peroxidation, Fe²⁺ content, glutathione peroxidase 4 (GPX4) expression, and mitochondrial morphology. Mevalonate pathway regulation was evaluated by western blotting, dual-luciferase assay, and quantification of squalene, coenzyme Q10 (CoQ10), and cholesterol. The role of growth differentiation factor 15 (GDF15) was validated using shRNA knockdown and overexpression DLD-1 cells* in vitro* and* in vivo*.

**Results:**

Arteannuin B showed significant anti-colorectal cancer activity both *in vitro* and *in vivo*. The proteomic analysis demonstrated that arteannuin B affected the mevalonate pathway and ferroptosis in DLD-1 cells, and strongly upregulated the expression of GDF15. Arteannuin B increased ROS, lipid peroxidation, malondialdehyde, and iron while decreasing GPX4 expression and causing mitochondrial shrinkage. Arteannuin B inhibited mevalonate pathway enzymes, particularly 3-hydroxy-3-methylglutaryl-CoA synthase 1 (HMGCS1), reducing squalene, CoQ10, and cholesterol. The knockdown of GDF15 weakened the inhibitory effect of arteannuin B on the mevalonate pathway and GPX4, and reduced the sensitivity of CRC cells to arteannuin B both *in vitro* and *in vivo*.

**Conclusion:**

Arteannuin B triggers ferroptosis-like cell death in CRC cells and suppresses xenograft growth, in association with inhibition of the mevalonate pathway. GDF15 contributes to arteannuin B-mediated suppression of HMGCS1 and GPX4 and to ferroptosis sensitivity.

## Introduction

Colorectal cancer is a type of cancer with a high incidence rate worldwide, and it is also a leading cause of cancer-related deaths 1. Researchers predict that the burden of colorectal cancer will increase to 3.2 million new cases and 1.6 million deaths by 2040 2. Systemic chemotherapy remains the primary treatment for advanced disease; however, commonly used agents, including 5-fluorouracil (5-FU), oxaliplatin, irinotecan, and capecitabine, frequently lead to acquired resistance following prolonged or repeated administration 3. Therefore, developing new therapeutic drugs is necessary and urgent. In recent years, targeted induction of ferroptosis has emerged as a promising strategy for colorectal cancer (CRC) treatment and may also offer new opportunities for diagnosis and prognosis.

Ferroptosis is a novel type of cell death mediated by iron ions and over-oxidized polyunsaturated fatty acids 4. Depletion of intracellular glutathione (GSH) and the deletion/inactivation of glutathione peroxidase 4 (GPX4) are thought to be the classical pathways causing ferroptosis 5. With gradually deepening research, the mevalonate pathway (MVP), a highly conserved cholesterol synthesis and lipid metabolism pathway in eukaryotic cells, had been confirmed to directly affect the process of ferroptosis. MVP is considered to protect cancer cells from ferroptosis through feedback regulation of cholesterol on coenzyme Q10 (CoQ10) and squalene 6 as well as the FSP1-CoQ10-NAD(P)H axis 7. And its intermediates isopentenyl pyrophosphate (IPP) 8,9, farnesyl pyrophosphate (FPP) and geranylgeranyl pyrophosphate (GGPP) 10,11 stabilize GPX4, while 7-Dehydrocholesterol (7-DHC) shields lipids from peroxidation 12. Therefore, the development of ferroptosis inducer and related drugs around MVP is becoming a valuable research field.

Growth differentiation factor 15 (GDF15), a member of the TGF-β superfamily, is involved in a variety of biological processes, including cell growth and proliferation, inflammation, and notably, the regulation of lipid metabolism 13. GDF15 has been widely reported to be associated with ferroptosis, but the molecular mechanism is still unclear. It is reported that GDF15 is significantly upregulated during erastin-induced ferroptosis in HT-1080 cells 14 and has been concluded to be a protective gene in the prognosis of colorectal cancer patients 15. Overexpression of GDF15 inhibited the proliferation and migration of clear cell renal cell carcinoma (ccRCC) and induced ferroptosis 16. In this article, we discovered a potential association between GDF15 and MVP. The GDF15-MVP axis that induces ferroptosis has been validated through *in vitro* and *in vivo* experiments, and these results may provide novel valuable information for the development of ferroptosis-based therapies.

*Artemisia annua* L. is a traditional herbal medicine that has been used for centuries to treat malaria 17, fever 18 and to induce sedation 19. Arteannuin B (CAS number: 50906-56-4) is a sesquiterpene lactone isolated from *A. annua*. Previous reports have revealed that arteannuin B has the effect of combating osteoporosis 20, anti-inflammation 21,22 as well as resisting SARS-CoV-2 23,24. Also, arteannuin B was found to show cytotoxicity to various human tumor cell lines *in vitro* 25. In the preliminary research, we found that arteannuin B enhanced the effectiveness of cisplatin on non-small cell lung cancer 26. But so far, the anti-colorectal cancer effect of arteannuin B and its potential mechanisms are not yet fully understood.

Recent research findings suggested that artemisinin compounds, such as artemisinin, dihydroartemisinin and artesunate, could exert anticancer activity by inducing cell ferroptosis 27. In this study, arteannuin B was first found to induce ferroptosis in CRC cells. Through systematic research on the anti-colorectal cancer effect of arteannuin B* in vitro* and *in vivo*, we demonstrated that arteannuin B induced ferroptosis in CRC cells through the GDF15/HMGCS1/GPX4 axis. This molecular mechanism differed from the reported induction of ferroptosis by artemisinin compounds, providing a new perspective on the biological process of ferroptosis and the treatment of colorectal cancer.

## Material and Methods

### Materials

Ferrostatin-1 (Fer-1), 3-methyladenine (3-MA), benzyloxycarbonyl-Val-Ala-Asp (OMe)-fluoromethylketone‌ (Z-VAD-FMK), and necrostatin-1 (Nec-1) were purchased from Selleck (Houston, USA). Anti-3-hydroxy-3-methylglutaryl-CoA reductase (HMGCR) antibody was purchased from ABclonal (Wuhan, China). Anti-HMGCS1 antibody, anti-GDF15 antibody, anti-FDFT1 antibody, anti-β-actin antibody and anti-GAPDH antibody were purchased from Proteintech (Wuhan, China). Anti-GPX4 antibody was purchased from Cell Signaling Technology (Danvers, USA). Radioimmunoprecipitation assay (RIPA) buffer, ROS detection kit, malondialdehyde (MDA) detection kit, and bicinchoninic acid (BCA) assay kit were purchased from Beyotime (Shanghai, China). Arteannuin B micelles and arteannuin B microspheres (ABMs) were prepared by Livzon Pharmaceutical Group Inc. Arteannuin B was isolated as previously reported 26.

### Cell lines

Human colorectal cancer cell lines (DLD-1, HCT 15, HCT 116, HT-29, SW480, SW620, RKO, COLO 320, and COLO 205), human normal intestinal epithelial cell line (HIEC-6), and human normal colon fibroblast cell line (CCD-18Co) were purchased from the National Collection of Authenticated Cell Cultures (Shanghai, China). The cells were cultured in Dulbecco's Modified Eagle's Medium (DMEM) or RPMI 1640, supplemented with 10% fetal bovine serum and penicillin-streptomycin (final concentration: 100 U/ml penicillin and 100 µg/ml streptomycin). All cells were incubated at 37 °C in a humidified atmosphere with 5% CO_2_.

### Cytotoxicity assay

Arteannuin B (6.25 to 100 µM) was incubated with cells for 24, 48, and 72 h, respectively. After incubation with methylthiazolyldiphenyl tetrazolium bromide solution (5 mg/ml) in the medium for 4 h, dimethyl sulfoxide (DMSO) was used to dissolve formazan. Subsequently, the OD_570nm_ value of each sample well was detected and the cell survival rate was calculated.

### Inhibition of ferroptosis

Different inhibitors were applied to block the cell death caused by arteannuin B. After the pretreatment of Fer-1 (2 µM, 4 h), 3-MA (100 µM, 2 h), Z-VAD-FMK (2 μM, 2 h), and Nec-1 (5 µM, 2 h), DLD-1 cells were then treated with arteannuin B (15 µM) and different inhibitors for 24 h. Cell viability was detected through MTT assay.

### ROS detection

ROS generation was detected by DCFH-DA staining. Briefly, DLD-1 cells were incubated with 5 μM DCFH-DA at 37 °C for 30 min after being treated with arteannuin B (7.5, 15, 30 µM) for 48 h. Quantitative analysis of intracellular ROS level was performed using flow cytometry according to the instructions of the ROS detection kit.

### Measurement of lipid peroxidation

DLD-1 cells were seeded in 6-well plates and treated with arteannuin B (3.75, 7.5, 15 µM) for 48 h. The cells were then collected and re-suspended in 2 μM C11-BODIPY^581/591^ (Cayman Chemical, MI, USA) for 30 min at 37 °C. Subsequently, cells were measured by flow cytometry. Quantification of data was performed with FlowJo 10.8.1.

### Detection of intracellular metabolites

DLD-1 cells were treated with arteannuin B for 48 h. Then, cells were lysed in RIPA buffer and the protein concentration was quantified by BCA assay kit. The intracellular MDA level was determined by the MDA detection kit. The intracellular iron level was determined by the iron assay kit (Sigma Aldrich, MO, USA). The content of squalene, cholesterol and CoQ10 in cell lysates was determined and quantified by squalene ELISA detection kit (Feiyue, Wuhan, China), total cholesterol assay kit (Solarbio, Shanghai, China) and CoQ10 ELISA detection kit (ZOKEYO, Wuhan, China).

### Transmission electron microscopy

Transmission electron microscopy was used to observe the morphological changes of mitochondria in DLD-1 cells after administration of arteannuin B. Briefly, DLD-1 cells were treated with arteannuin B at a concentration of 15 µM for 48 h. Cells were collected and fixed in a fixation solution containing 2.5% glutaraldehyde. Subsequently, the samples were sent to Wuhan Servicebio Technology Co., Ltd (Wuhan, China) for dehydration, embedding, sectioning, and staining processes. Transmission electron microscope was used to capture the pictures.

### Western blotting analysis

DLD-1 cells were treated with arteannuin B for 48 h and lysed in RIPA buffer. The cell lysates were centrifuged and protein concentration was measured using a BCA assay kit. Equal amounts of protein were separated by SDS-PAGE. The proteins were then transferred onto a polyvinylidene fluoride (PVDF) membrane and blocked with bovine serum albumin to prevent nonspecific binding. Next, the membrane was incubated with a primary antibody specific to the target protein, followed by an HRP-conjugated secondary antibody. Finally, the target protein was visualized using an enhanced chemiluminescence kit and chemiluminescence imaging system.

### Real-time quantitative PCR analysis

Total RNA was isolated from cells or tissue samples using Trizol reagent (Beyotime, Shanghai, China) strictly following the manufacturer's instructions. During the extraction process, the volume of lysis buffer was carefully controlled, and only the upper aqueous phase was collected to avoid genomic DNA contamination caused by organic phase carry-over. The concentration and quality of the extracted total RNA were assessed using a NanoD2000C (Thermo, USA) to verify RNA purity.

Synthesis of cDNA was performed by RT-PCR from 1000 ng of total RNA using the Hifair® Ⅱ 1st Strand cDNA Synthesis SuperMix for qPCR (Yeasen, Shanghai, China), which contains a gDNA digester that specifically removes residual genomic DNA from RNA samples. To further ensure no genomic DNA amplification during the PCR step, a no-RT negative control was set for each group.

The sample mixture was prepared according to the TransStart® Top Green qPCR SuperMix (TransGen, Beijing, China) instructions. Then, RT-qPCR experiment was performed by LightCycler® 480 (Roche, Basel, Switzerland). All reactions were normalized to GAPDH level, and 2^-ΔΔCt^ method was used for data analysis.

### Proteomic analysis

Proteomic analysis was supported by Shanghai Applied Protein Technology Co. Ltd (Shanghai, China). Briefly, DLD-1 cells were incubated with or without 15 µM arteannuin B for 48 h. Cell lysates were then collected and subjected to tandem mass tag (TMT) relative quantitative proteomic analysis. The T/C value was defined as the difference in protein expression multiples between the arteannuin B treatment group and the normal control group. Proteins with a T/C value outside 0.83 to 1.2 and *p* < 0.05 were confirmed as significantly differentially expressed proteins (SDEPs). Bioinformatics analysis was performed on the identification results.

### Survival Analysis

The Kaplan-Meier method was employed to construct overall survival curves for colorectal cancer patients using the GEPIA2 tool. The discrepancy in survivals was assessed through the utilization of the log-rank test, alongside the calculation of the HRs (hazard ratio with 95% confidence interval). The core data for GEPIA survival analysis were derived from two major international public databases: the Cancer Genome Atlas (TCGA) and the Genotype-Tissue Expression (GTEx).

### Plasmid construction and cell transfection

GDF15 specific shRNA plasmids (shGDF15: 5'-AGACTCCAGATTCCGAGAGTT-3'), GDF15 overexpression plasmids, GPX4 overexpression plasmids, lentiviral plasmids, sterol regulatory element-binding protein 2 (SREBP2) plasmid and control plasmid, HMGCS1 promoter plasmid and control plasmid, and marine luciferase plasmid were purchased from Transheepbio (Shanghai, China).

HEK293T cells were incubated with lentiviral vectors, psPAX2 and pMD2G vectors, PEI (4 μg/ml) for virus production. After two days of normal culture of cells, the cells were then infected with the virus for 24 h. Puromycin (3 μg/ml) was used to select stably transduced cells for 1 week.

For the dual-luciferase reporter assay, DLD-1 cells were seeded at a density of 1×10⁶ cells per well in a six-well plate. When the cell density reached 70%, transient transfection was used to transfer the HMGCS1 promoter plasmid and promoter control plasmid. The detection of the binding of SREBP2 and HMGCS1 promoter before and after 15 μM arteannuin B was carried out using glow type dual luciferase reporter gene detection kit (Yeasen, Shanghai, China). A blank control group was set to subtract the background, and the expression fold was calculated as (firefly luciferase value of the experimental group/Renilla luciferase value)/(corresponding ratio of the control group).

### *In vivo* anti-colorectal cancer experiment of arteannuin B

DLD-1 cells (5×10⁷ cells/mL) were subcutaneously injected (100 μL) into left axillary regions of male BALB/c nude mice (6-7 weeks old). When tumors reached approximately 70 mm³, mice were randomized into six groups receiving daily treatments for 21 days: model group (10 ml/kg normal saline), blank micelles group (blank micelles), arteannuin B micelles group (20 mg/kg arteannuin B micelles), vehicle group (1 ml/kg DMSO), arteannuin B group (20 mg/kg arteannuin B), and 5-FU group (20 mg/kg 5-FU). During the administration period, the tumor volume of mice in each group was detected every other day. Post-treatment, mice were euthanized for organ collection (liver, kidneys, spleen, heart) and histopathological analysis. Tumor tissues were cryopreserved (-80°C) and formalin-fixed. GPX4 expression in tumor specimens was assessed by immunohistochemistry (IHC).

### *In vivo* experiments identifying GDF15 as a key target protein

DLD-1^WT^ and DLD-1^shGDF15^ cells (2.0×10⁷ cells/mL) were subcutaneously injected (100 μL) into left axillary regions of male BALB/c nude mice (6-7 weeks old). Mice with tumors approximately 70 mm³ were randomized into six groups: DLD-1^WT^/Model group (10 ml/kg normal saline), DLD-1^WT^/Vehicle group (blank microspheres), DLD-1^WT^/ABMs group (140 mg/kg ABMs), DLD-1^shGDF15^/Model group (10 ml/kg normal saline), DLD-1^shGDF15^/Vehicle group (blank microspheres), DLD-1^shGDF15^/ABMs group (140 mg/kg ABMs), treated weekly for 4 weeks. During the administration period, the tumor volume of mice in each group was detected every other day. Post-treatment, mice were euthanized for organ collection (liver, kidneys, spleen, heart) and histopathological analysis. Tumor tissues were cryopreserved (-80°C) and formalin-fixed. GPX4 expression in tumor specimens was assessed by IHC, while organizational lysate was analyzed for MDA and cholesterol levels.

### Statistical analysis

Statistical analysis was performed using SPSS (Version 25.0) and GraphPad Prism (Version 9.5.1). Graphs were established using GraphPad Prism (Version 9.5.1) and ImageJ (Version 1.5.4). Results were expressed as mean ± standard deviation (SD), n ≥ 3. Two-tailed Student's t-test was used to assess differences between two groups, and one-way analysis of variance (ANOVA) followed by Sidak's multiple comparisons test was used to count differences between multiple groups. Statistical significance was considered for *p* < 0.05.

## Results

### Anti-colorectal cancer effects of arteannuin B *in vitro* and *in vivo*

**Figure 1:**
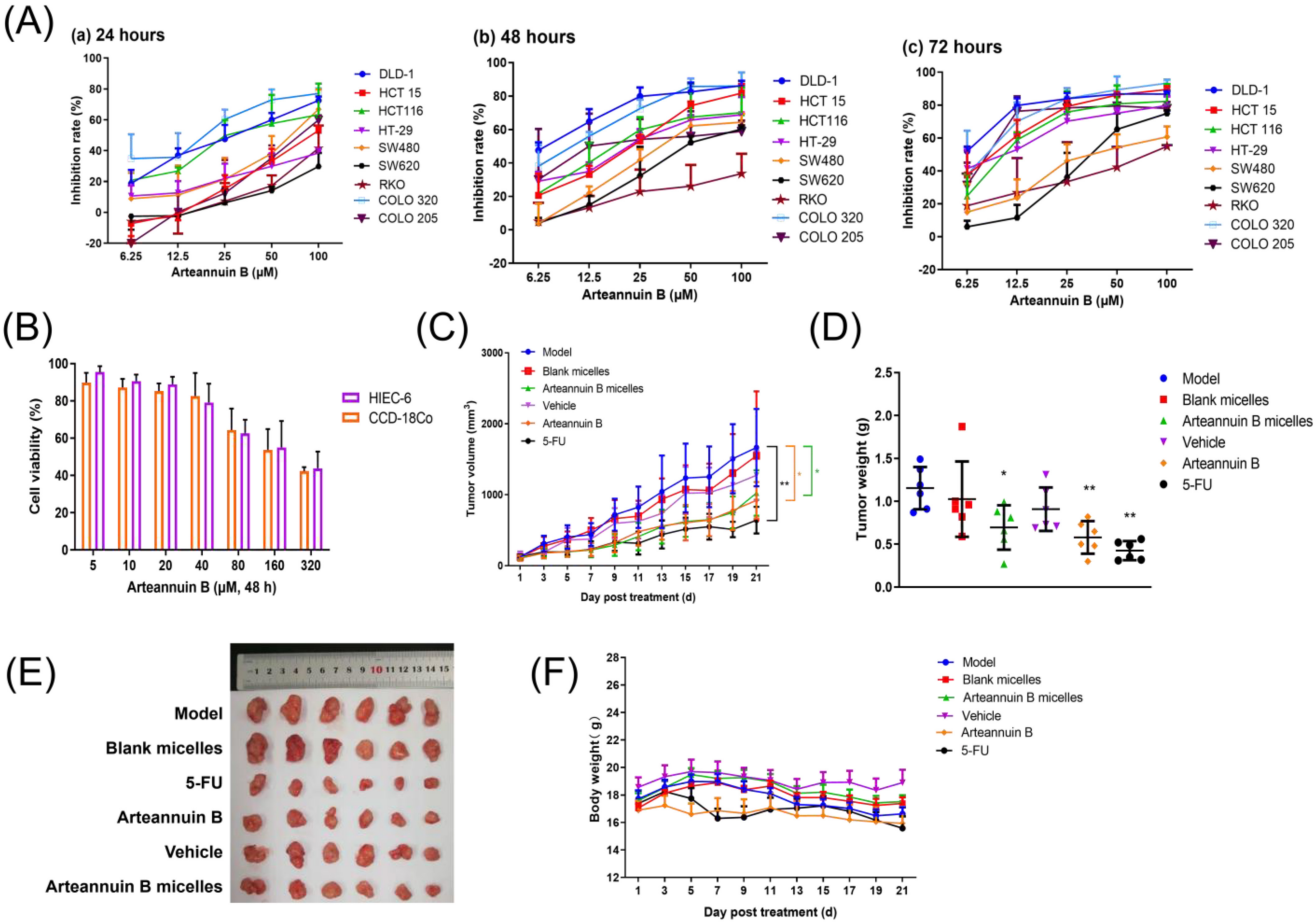
** Arteannuin B showed anti-colorectal cancer effects *in vitro* and *in vivo*.** (A) Anti-proliferative effects of arteannuin B on nine human CRC cells at (a) 24 h, (b) 48 h, and (c) 72 h (*n* = 3). (B) Effect of arteannuin B on viability of CCD-18Co cells and HIEC-6 cells (*n* = 3). (C-D) Effect of arteannuin B on tumor volume and tumor weight of nude mice xenografted with DLD-1 cells (*n* = 6). (E) Pictures of tumor tissues. (F) Changes of the body weight of animals (*n* = 6). Data are presented as mean ± SD;^ *^p < 0.05, ^**^p < 0.01 vs. model group.

The results of the cytotoxicity experiment showed that arteannuin B exhibited potent anti-proliferative effects across a panel of 9 human colorectal cancer cell lines in a concentration- and time-dependent manner (Figure 1A), suggesting its broad-spectrum activity against CRC cells. After 72 h of treatment on DLD-1, COLO 205, COLO 320, HCT 15 and HT-29 cells, the IC_50_ values of arteannuin B were all less than 10 µM (Table S1). Based on the screening results, DLD-1 was identified as the most responsive cell line to arteannuin B and was therefore chosen as the primary model for subsequent mechanistic investigations.

When arteannuin B was applied to human normal colon fibroblasts CCD-18Co and human normal intestinal epithelial cells HIEC-6 for 48 h, the IC_50_ values of arteannuin B were 165.15 ± 49.56 μM and 228.49 ± 72.94 μM, respectively (Figure 1B). Within the commonly used concentration range from 3.75 to 15 μM in this study, arteannuin B showed no significant effect on the cell viability of normal colon cells (the cell viability is greater than 85%), but selectively inhibited the proliferation of CRC cells.

The DLD-1 cell xenograft tumor model was established to detect the *in vivo* anti-tumor effect of arteannuin B. The experimental results demonstrated that 20 mg/kg arteannuin B significantly inhibited the growth of xenograft tumors (Figure 1C) with the inhibition rate of 49.71% (Figure 1D-E). After 21 d of arteannuin B treatment, the body weight of the animals in the treatment group showed no significant differences compared to the model group (Figure 1F), and the organ indices of mice remained unaffected (Table 1). These data showed that arteannuin B did not show obvious toxic effects under experimental conditions.

### Proteomic analysis of the anti-colorectal cancer effect of arteannuin B

**Figure 2:**
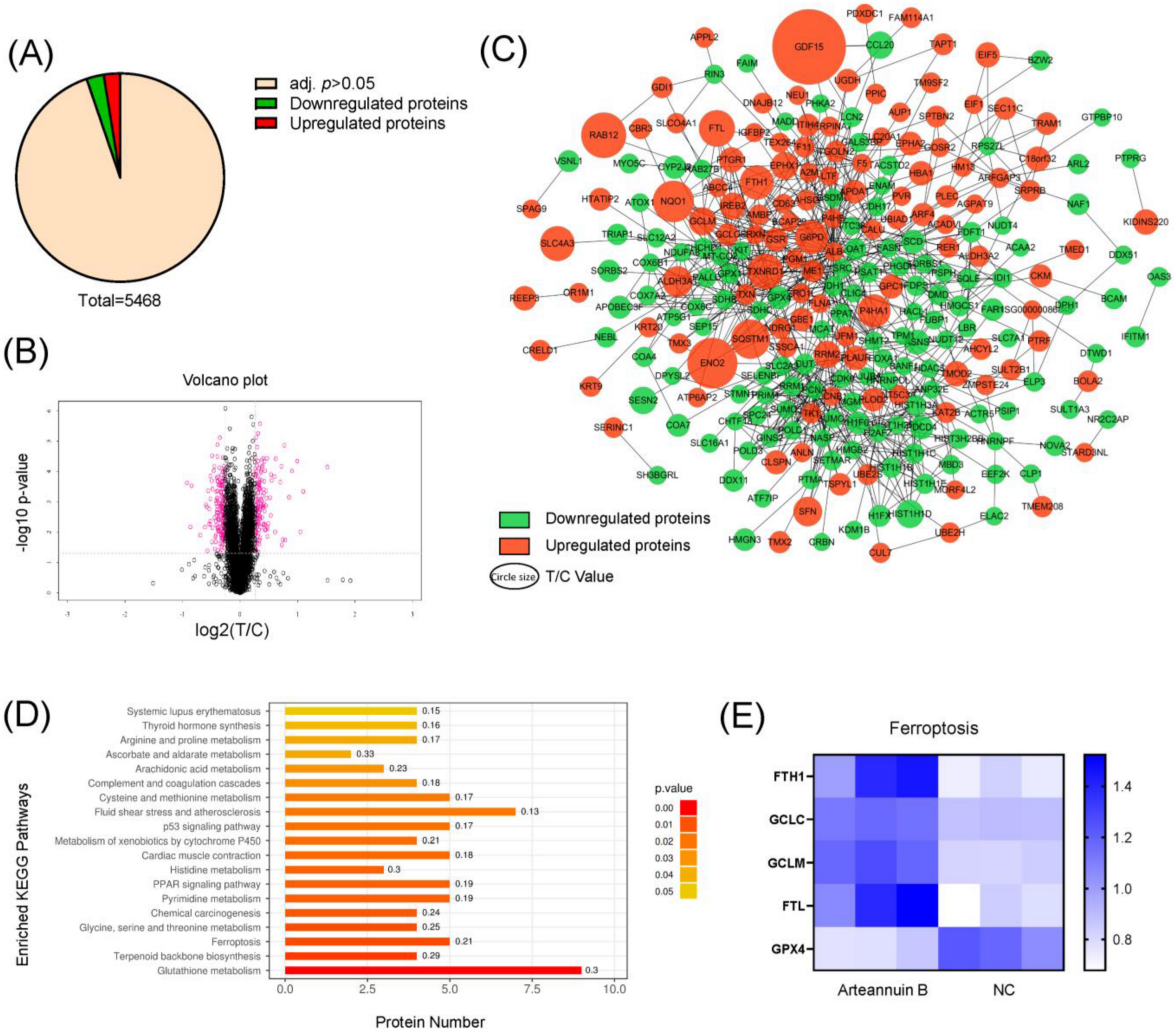
** Proteomic analysis of the anti-colorectal cancer effect of arteannuin B.** (A) Overview of proteomics analysis results. (B) Volcano plot of SDEPs. (C) Visualization of SDEPs. (Green: downregulated proteins; Red: upregulated proteins; Size of the circle: T/C value). (D) KEGG analysis of SDEPs. (E) SDEPs enriched in the ferroptosis signaling pathway.

After treating DLD-1 cells with arteannuin B for 48 h, we extracted total protein for TMT-based quantitative proteomic analysis. A total of 5468 proteins were identified (Figure 2A). The T/C value was defined as the difference in protein expression multiples between the arteannuin B treatment group and the normal control group. When the T/C value was not within the range of 0.83 to 1.2 times and the p-value was less than 0.05, the protein was identified as being differentially expressed (Figure 2B). A total of 144 differentially upregulated proteins and 152 differentially downregulated proteins were identified. Among them, the most significantly upregulated protein is GDF15 (T/C=2.869102), and the most significantly downregulated protein is histone H1.3 (encoded by *HIST1H1D*) (T/C=0.527179) (Figure 2C). The results of Kyoto Encyclopedia of Genes and Genomes (KEGG) pathway enrichment analysis showed that SDEPs were mainly enriched in biological pathways related to glutathione metabolism, terpenoid backbone biosynthesis (mevalonate pathway), and ferroptosis pathway (Figure 2D). The proteins related to the ferroptosis pathway were involved in ferritin heavy chain (FTH1), glutamate-cysteine ligase catalytic subunit (GCLC), glutamate-cysteine ligase regulatory subunit (GCLM), ferritin light chain (FTL) and GPX4 (Figure 2E). Among them, FTH1, GCLC, GCLM, and FTL were observed to be significantly up-regulated, and these increases are likely to represent an active cellular defense aimed at limiting ferroptosis progression 28. In contrast, the decreased expression of GPX4 is one of the most emblematic molecular events of ferroptosis.

### Arteannuin B induced ferroptosis in CRC cells

**Figure 3:**
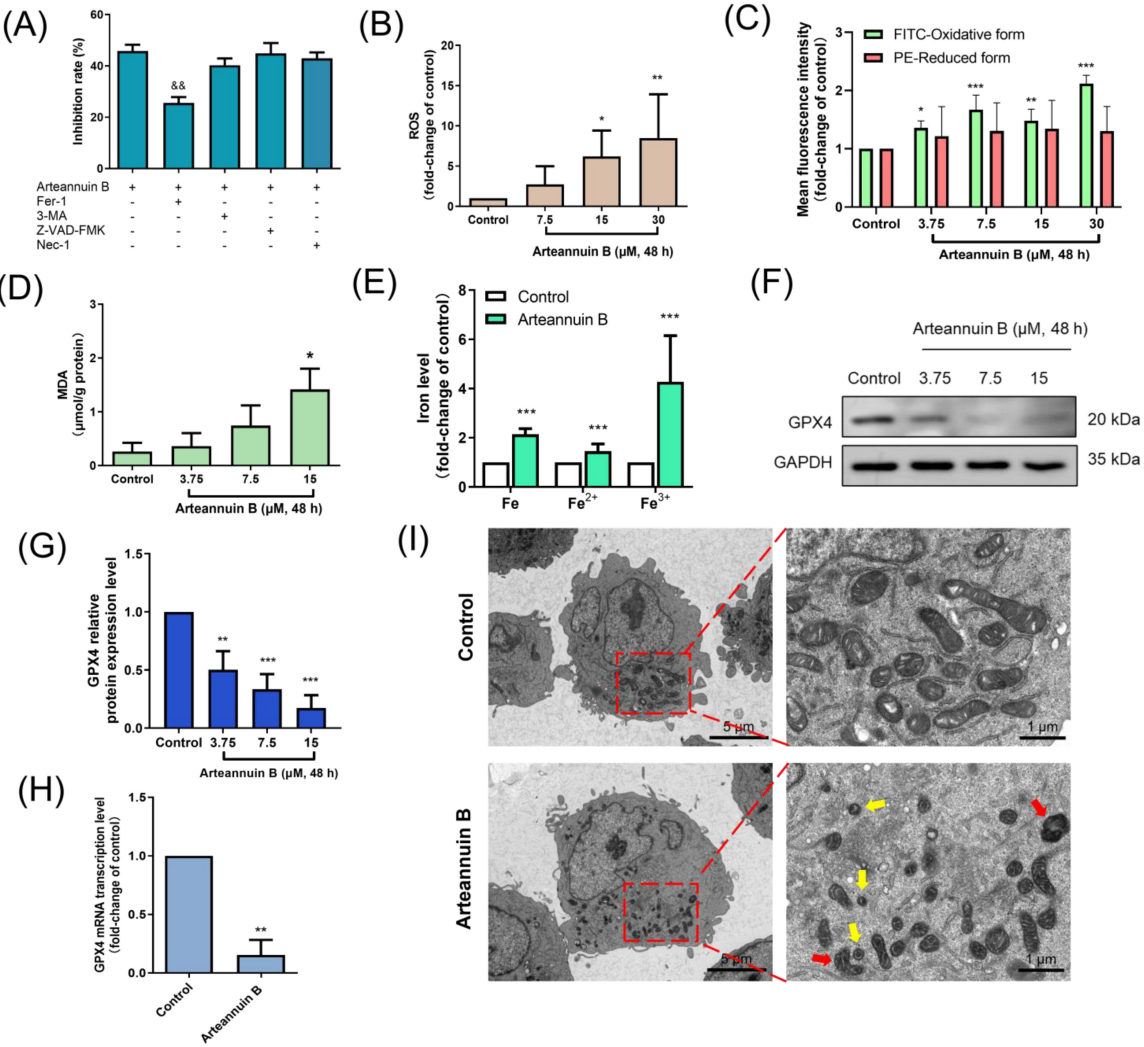
** Arteannuin B induced ferroptosis in CRC cells.** (A) Influence of cell death inhibitors on the anti-proliferative effect of arteannuin B against DLD-1 cells. (B-D) Effects of arteannuin B on ROS, lipid peroxidation, MDA levels in DLD-1 cells. (E) Effects of arteannuin B on iron ions content in DLD-1 cells. (F-G) Western blotting images and quantitative analysis showed the effects of arteannuin B on GPX4 protein expression levels in DLD-1 cells. (H) Effects of arteannuin B on mRNA transcription level of GPX4 in DLD-1 cells. (I) Effects of arteannuin B on mitochondrial morphology of DLD-1 cells. In the control group, mitochondria are elongated rods with parallel cristae (upper panel). In the arteannuin B-treated group, mitochondria are smaller and rounded (yellow arrows), with a denser matrix (darker staining), discontinuous outer membrane, and loss of cristae (red arrows). Scale bar = 5 μm, 1 μm. Data are presented as mean ± SD, *n* = 3;^ &&^*p* < 0.01 vs. arteannuin B group without inhibitor treatment;^ *^p < 0.05, ^**^p < 0.01,^ ***^p < 0.001 vs. control group.

To determine whether arteannuin B induced ferroptosis in CRC cells, DLD-1 cells were pretreated with autophagy inhibitor (3-MA), apoptosis inhibitor (Z-VAD-FMK), necroptosis inhibitor (Nec-1), and ferroptosis inhibitor (Fer-1), respectively. The results showed that only the ferroptosis inhibitor could significantly relieve the inhibitory effect of arteannuin B on DLD-1 cells (Figure 3A).

Out-of-control membrane lipid peroxidation is a critical step in inducing ferroptosis 29. It was observed that arteannuin B significantly increased the content of ROS (Figure 3B) and the level of lipid peroxidation (Figure 3C) in DLD-1 cells. The level of MDA, a lipid peroxidation product, was also detected to increase after the treatment of arteannuin B (Figure 3D). These results indicated that arteannuin B disrupted the intracellular redox balance and induced lipid peroxidation in DLD-1 cells. In addition, arteannuin B leaded to an increase in iron ion level within CRC cells (Figure 3E). The iron accumulation caused by arteannuin B showed an increase in total intracellular iron content, rather than an increase only in Fe^2+^ content.

As mentioned earlier, GPX4 is a key protein in cells that resists ferroptosis. After the DLD-1 cells were treated with arteannuin B for 48 h, the protein expression level of GPX4 was significantly downregulated (Figure 3F-G), and the mRNA transcription level of GPX4 also showed a significant decrease (Figure 3H). In addition, arteannuin B also downregulated the protein expression level of GPX4 in the DLD-1 cells xenograft tumor tissues (Figure S1). These data indicated that GPX4 protein was significantly inhibited in DLD-1 cells after administration of arteannuin B.

The morphological changes of cell mitochondria are another important indicator of ferroptosis 30. DLD-1 cells treated with 15 μM arteannuin B were examined for alterations in organelle morphology using transmission electron microscopy. As shown in Figure 3I, in normally cultured DLD-1 cells, mitochondria appeared as elongated rods with parallel-arranged cristae. After arteannuin B treatment, the mitochondrial volume was markedly reduced and became round (yellow arrows). The matrix showed high electron density (darkened), the outer membrane was discontinuous in places, and the cristae had disappeared (red arrows). Our results demonstrated that arteannuin B inhibited the proliferation of CRC cells by inducing ferroptosis.

### GDF15 was identified as a key factor in arteannuin B-induced ferroptosis

Based on the data of proteomic analysis, GDF15 was found to be the most significantly upregulated protein in DLD-1 cells after the administration of arteannuin B (Figure 2C). In previous studies, a multitude of natural products and active ingredients of traditional Chinese medicine have been documented to elicit anti-neoplastic actions against colorectal carcinoma, potentially through the modulation of GDF15 and its associated signaling cascades 31,32. Our subsequent research endeavors aimed to elucidate whether GDF15 represents a molecular target of arteannuin B in its anti-colorectal cancer activity, and to delineate the mechanistic role of GDF15 in arteannuin B-induced ferroptosis.

**Figure 4:**
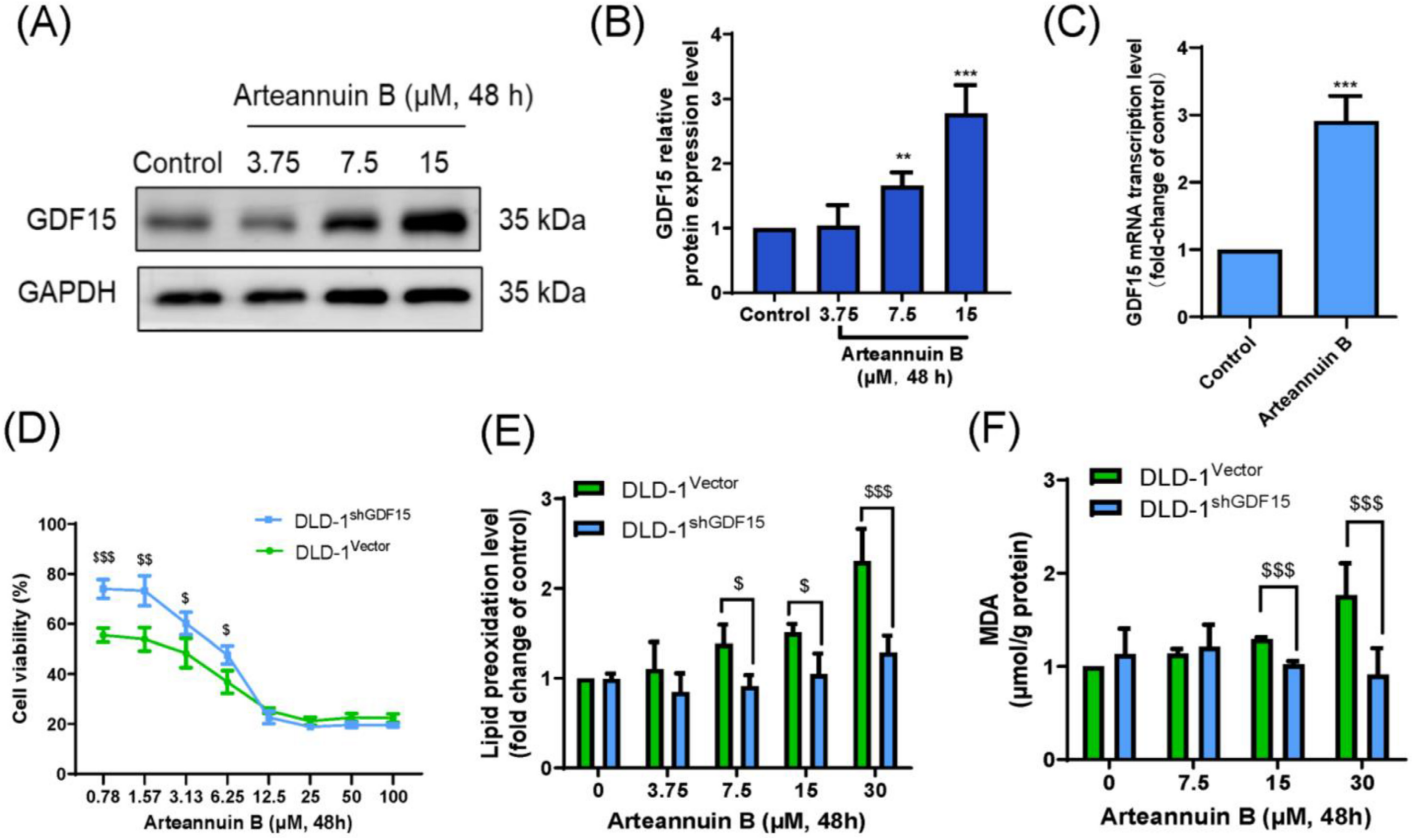
** GDF15 was identified as a key factor in arteannuin B-induced ferroptosis.** (A-B) Western blotting images and quantitative analysis showed the effects of arteannuin B on GDF15 protein expression level in DLD-1 cells. (C) Effect of arteannuin B on the mRNA transcription level of GD15 in DLD-1 cells. (D) Anti-proliferative effects of arteannuin B on DLD-1^Vector^ and DLD-1^shGDF15^ cells. (E-F) Effects of arteannuin B on lipid peroxidation and MDA levels in DLD-1^Vector^ and DLD-1^shGDF15^ cells. Data are presented as mean ± SD, *n* = 3;^ **^p < 0.01,^ ***^p < 0.001 vs. control group;^ $^*p* < 0.05, ^$$^*p* < 0.01,^ $$$^*p* < 0.001 vs. DLD-1^Vector^ cells.

Consistent with the results of proteomic analysis, arteannuin B caused an increase in the protein expression level of GDF15 in DLD-1 cells (Figure 4A-B) and also upregulated the mRNA transcription level of GDF15 (Figure 4C).

Subsequently, the impact of GDF15 deficiency on the anti-colorectal cancer effect of arteannuin B was studied. We silenced GDF15 in DLD-1 cells using shRNA (Figure S2A-B). Knockdown of GDF15 reduced the sensitivity of DLD-1 cells to arteannuin B, significantly increasing cell viability (Figure 4D). Compared with DLD-1^Vector^ cells, the intracellular lipid peroxidation level of DLD-1^shGDF15^ cells was significantly reduced (Figure 4E). Similarly, the production rate of MDA also decreased significantly (Figure 4F).

These data suggested that GDF15 was a key factor in arteannuin B-induced ferroptosis in DLD-1 cells. In the absence of GDF15, arteannuin B could not successfully induce intracellular lipid peroxidation, and led to significantly enhanced resistance of DLD-1 cells to arteannuin B.

### Arteannuin B significantly inhibited the mevalonate pathway in colorectal cancer

As mentioned earlier, the metabolic flux of MVP is crucial for maintaining the normal function of GPX4 and resisting ferroptosis in cells. The results of KEGG analysis showed that SDEPs were remarkably enriched in MVP (Figure 2D), which is the core metabolic pathway of cholesterol synthesis associated with terpenoid backbone biosynthesis.

**Figure 5:**
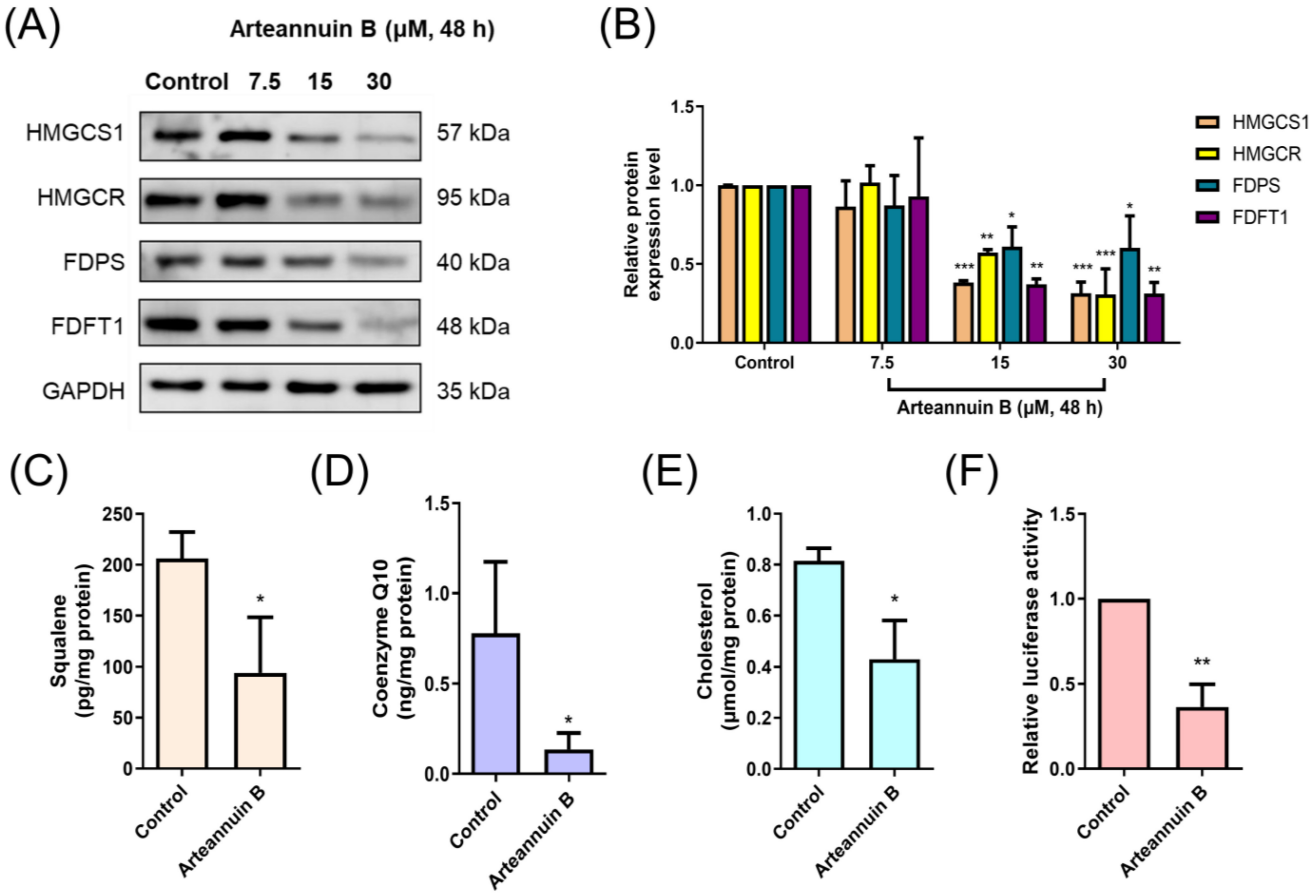
** Arteannuin B inhibited the mevalonate pathway in colorectal cancer.** (A-B) Western blotting images and quantitative analysis showed the effects of arteannuin B on MVP-related protein expression levels in DLD-1 cells. (C-E) Effect of arteannuin B on MVP products in DLD-1 cells, squalene (C), CoQ10 (D) and cholesterol (E). (F) The effect of arteannuin B on the binding of HMGCS1 and SREBP2 (dual luciferase reporter gene assay). Data are presented as mean ± SD, *n* = 3;^ *^p < 0.05,^ **^p < 0.01,^ ***^p < 0.001 vs. control group.

As shown in Figure 5A-B, arteannuin B significantly downregulated the expression of various enzymes in the MVP, including HMGCS1, HMGCR, farnesyl diphosphate synthase (FDPS), and FDFT1. Among them, HMGCS1 and HMGCR are considered as key enzymes of MVP, which directly regulate the flux of MVP 33. On the other hand, arteannuin B treatment significantly reduced the levels of mevalonate pathway products, including squalene, CoQ10, and cholesterol, in DLD-1 cells (Figure 5C-E). Arteannuin B strongly inhibited the expression of key regulatory enzymes of MVP and led to the reduction of its products. SREBP2 is a classic cholesterol regulatory factor that can directly regulate the gene expression of the mevalonate pathway 34. The dual luciferase reporter gene experiment showed that arteannuin B can weaken the binding of SREBP2 and HMGCS1(Figure 5F). These results indicate that arteannuin B can significantly inhibit the mevalonate pathway in CRC cells.

### Arteannuin B induced ferroptosis via GDF15/HMGCS1/GPX4 axis

**Figure 6:**
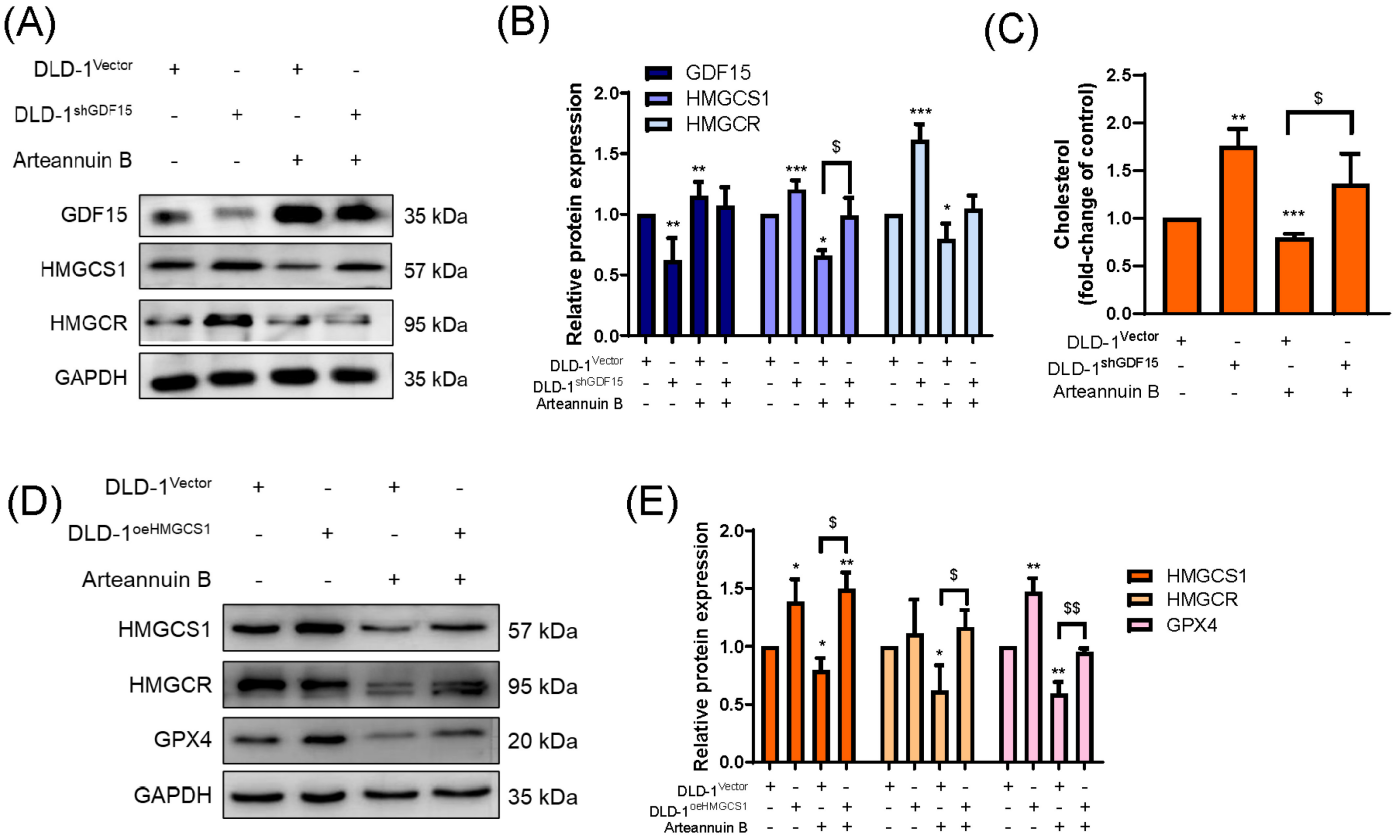
** Arteannuin B induced ferroptosis via GDF15/HMGCS1/GPX4 axis.** (A-B) Western blotting images and quantitative analysis showed the effects of GDF15 knockdown on the expression levels of MVP-related proteins. (C) Effect of GDF15 knockdown on the cholesterol level in DLD-1^Vector^ and DLD-1^shGDF15^ cells. (D-E) Western blotting images and quantitative analysis showed the effects of arteannuin B on the protein expression level in DLD-1^oeHMGCS1^ cells. Data are presented as mean ± SD, *n* = 3;^ *^p < 0.05,^ **^p < 0.01,^ ***^p < 0.001 vs. control group; ^$^*p* < 0.05,^ $$^*p* < 0.01 vs. DLD-1^Vector^ cells.

It has been reported that GDF15 can regulate cholesterol synthesis in esophageal cancer cells through the sterol regulatory element-binding protein cleavage-activating protein (SCAP)/SREBP2 axis 35. Therefore, we speculated that GDF15 also has a regulatory effect on the cholesterol synthesis pathway in CRC cells. We examined the changes in expression levels of MVP-related enzymes in DLD-1^shGDF15^ cells. The inhibitory effect of arteannuin B on HMGCS1, instead of HMGCR, was significantly weakened in DLD-1^shGDF15^ cells (Figure 6A-B). Meanwhile, the inhibitory effect of arteannuin B on cholesterol in DLD-1 cells was reversed when GDF15 was knocked down (Figure 6C). In the absence of GDF15, arteannuin B lost its inhibitory activity against MVP. Knockdown of GDF15 caused significant upregulation of HMGCS1.

Subsequently, DLD-1 cells with overexpression of HMGCS1 protein were constructed (Figure S2C-D). In DLD-1^oeHMGCS1^ cells, we observed that the inhibitory effect of arteannuin B on HMGCR and GPX4 was significantly weakened (Figure 6D-E). This result proved that HMGCS1 was a key factor in the regulation of MVP by arteannuin B, and arteannuin B achieved the synthesis inhibition of GPX4 protein by inhibiting HMGCS1.

To further confirm the regulatory effect of GDF15 on MVP, we successfully constructed DLD-1^oeGDF15^ cells (Figure S2E-F) and examined the effects of GDF15 on MVP and GPX4. The experimental results showed that high-level of GDF15 inhibited the expression of two key enzymes of MVP (HMGCS1 and HMGCR) as well as GPX4 protein (Figure S3A-B). Besides, overexpression of GDF15 formed a synergistic effect with arteannuin B, further inhibiting the expression of the above three proteins.

Our experimental result demonstrated that the arteannuin B-induced ferroptosis and the inhibitory effect of arteannuin B on MVP were all dependent on the presence of GDF15 protein. It supported that arteannuin B induced ferroptosis in CRC cells through the GDF15/HMGCS1/GPX4 signaling axis.

### Knockdown of GDF15 weakened the anti-colorectal cancer effect of arteannuin B *in vivo*

**Figure 7:**
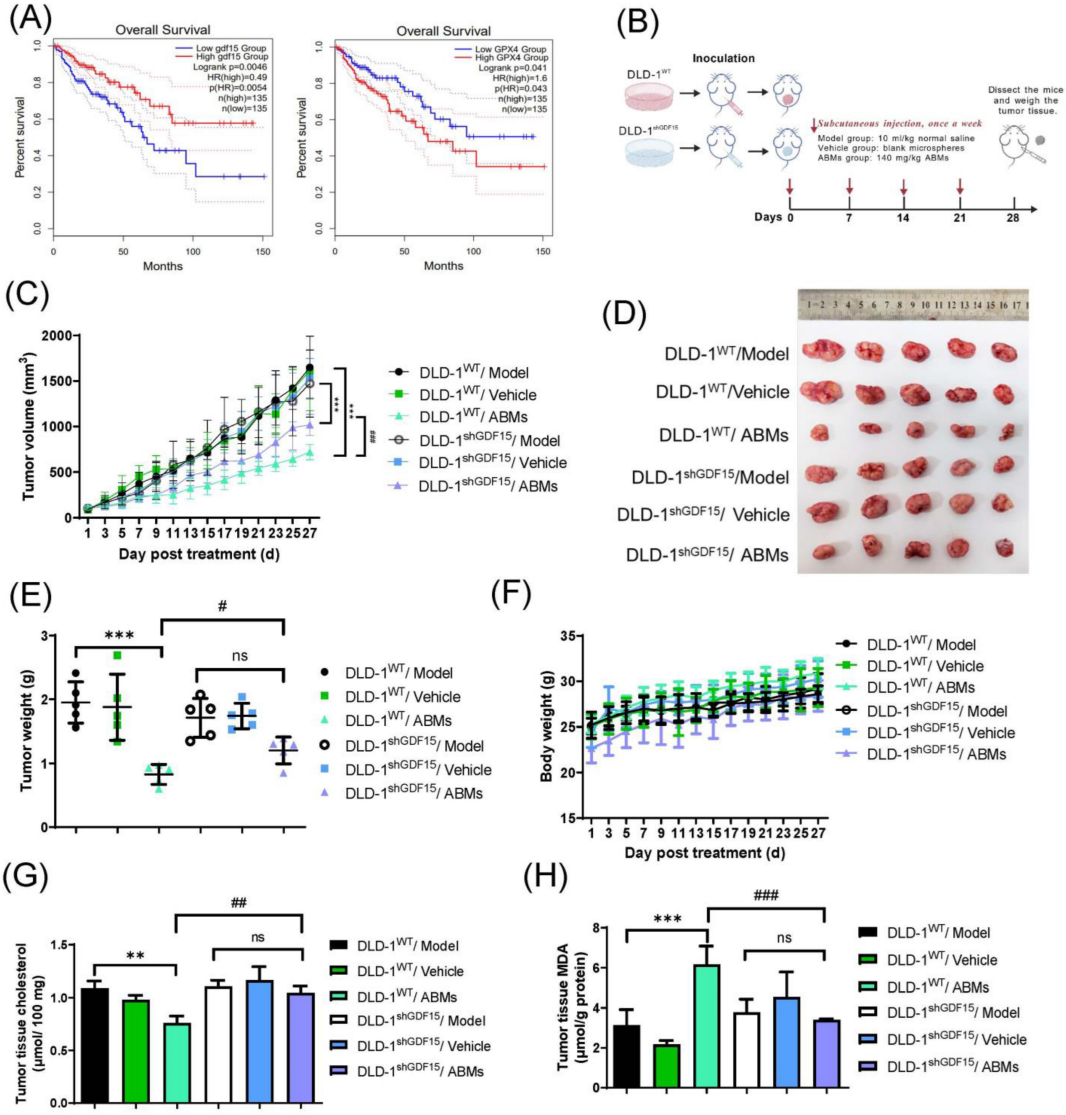
** Knockdown of GDF15 weakened the anti-colorectal cancer effect of arteannuin B *in vivo*.** (A) Kaplan-Meier analysis of survival statistic in patient data from public databases. The dotted lines represent the 95% confidence intervals (95% CI) for the survival estimates. (B) Schematic diagram of animal experiment. (C) Tumor growth curves (*n* = 5). (D) Pictures of tumor tissues. (E) Tumor weight (*n* = 5). (F) Curve of animal weight changes (*n* = 5). (G-H) Cholesterol and MDA levels in tumor tissues (*n* = 3). Data are presented as mean ± SD;^ *^p < 0.05, ^**^p < 0.01,^ ***^p < 0.001 vs. model group;^ #^*p* < 0.05, ^##^*p* < 0.01,^ ###^*p* < 0.001 vs. DLD-1^WT^ cells; ns, no significant.

GDF15 has been recognized as a potent suppressor of oncogenic processes, with demonstrated direct interactions with a spectrum of tumor-suppressor genes, including P53, early growth response protein 1 (EGR-1), and peroxisome proliferator-activated receptor gamma (PPARγ). These interactions are posited to contribute to the inhibitory effects of GDF15 on the proliferation of neoplastic cells 36,37. Using GDF15 and GPX4 expression as variables, we collected the data of patient samples from public databases and conducted Kaplan-Meier survival analyses on the included patient cohort. The GEPIA2 tool was used to construct overall survival curves 38. The results indicated that high level of GDF15 and low level of GPX4 in tumor tissues typically imply longer median survival (Figure 7A).

Next, we investigated the impact of GDF15 knockdown on the *in vivo* anti-colorectal cancer efficacy of arteannuin B (Figure 7B). ABMs were prepared as our previous report 39. The results of xenograft tumor experiment indicated that ABMs significantly inhibited the growth of DLD-1^WT^ and DLD-1^shGDF15^ cells* in vivo* (Figure 7C-D). DLD-1^WT^/Model group and DLD-1^shGDF15^/Model group showed similar growth curves indicating that knockdown of GDF15 did not affect the growth of DLD-1 xenograft tumors. During the administration cycle, the loss of GDF15 protein significantly reduced the sensitivity of DLD-1 cells to ABMs, weakening the inhibition of xenograft tumor growth by ABMs (Figure 7C, DLD-1^ shGDF15^/ABMs group *vs* DLD-1^WT^/ABMs group). After the tumor tissue was dissected and the tumor inhibition rate was calculated, the results showed that the inhibitory rates of ABMs on DLD-1^WT^ and DLD-1^shGDF15^ xenograft tumors were 57.64% (DLD-1^WT^/ABMs group) and 29.71% (DLD-1^shGDF15^/ABMs group), respectively (Figure 7E). The *in vivo* anti-colorectal cancer effect of arteannuin B was greatly inhibited in the absence of GDF15.

During the administration period, there were no significant changes between ABMs groups and model groups in the body weight (Figure 7F) and the organ indices of mice (Table 2).

IHC analysis was used to examine the protein expression of GPX4 in tumor tissues. As shown in Figure S4, arteannuin B significantly inhibited the protein expression of GPX4 in DLD-1^WT^ xenograft tumor tissues. However, the protein expression of GPX4 was restored in DLD-1^shGDF15^ xenograft tumor tissues.

Similarly, we also observed that the level of cholesterol, the end-product of MVP, was significantly inhibited by arteannuin B in DLD-1^WT^ xenograft tumor tissues. This inhibitory effect was eliminated in DLD-1^shGDF15^ xenograft tumor tissues (Figure 7G). On the other hand, arteannuin B treatment induced an increase in the content of lipid peroxidation product MDA in tumor tissues, and this phenomenon was alleviated by knockdown of GDF15 (Figure 7H).

The results above demonstrated that GDF15 is a key protein in arteannuin B-induced ferroptosis in CRC cells *in vitro* and *in vivo*. The anti-colorectal cancer effect of arteannuin B was greatly weakened after GDF15 was knocked down.

## Discussion

At present, the standard treatments for colorectal cancer include surgery, radiotherapy, and chemotherapy 40. Nevertheless, the issue of drug resistance has underscored an urgent call for the development of novel therapeutic agents. In this study, we evaluated the anti-tumor activity of arteannuin B, a sesquiterpene lactone in *Artemisia annua*, both* in vitro* and *in vivo*. For the first time, we systematically demonstrated that arteannuin B induces ferroptosis in CRC cells.

Previous studies have established artemisinin compounds as ferroptosis inducers, which primarily regulate Fe²⁺ metabolism and oxidative stress 41, while alternative mechanisms include endoplasmic reticulum stress 42, P53 signaling 43, regulation of SREBP2 nuclear localization 44. Our findings indicated that arteannuin B was more likely to disrupt intracellular lipid metabolism (Figure 3B-D), rather than directly perturb Fe^2+^ metabolism (Figure 3E). We proposed a novel mechanism by which arteannuin B induces ferroptosis in CRC cells through the GDF15/HMGCS1/GPX4 axis.

GDF15 was identified as the most significantly upregulated protein after treatment with arteannuin B (Figure 2C). When GDF15 was knocked down, arteannuin B-induced ferroptosis was weakened both *in vitro* and *in vivo* (Figure 4D-F and Figure 7C-E), and tumor tissue cholesterol no longer decreased (Figure 7G), indicating that GDF15 is a necessary upstream switch for inhibiting the mevalonate pathway and subsequent ferroptosis.

Recent studies have reported conflicting roles of GDF15 in ferroptosis. In microsatellite instability-high colorectal cancer (MSI-H CRC), GDF15 suppression enhances ferroptosis sensitivity 45, whereas in ccRCC, GDF15 overexpression promotes ferroptosis 16. Another study showed that GDF15 overexpression alleviates myocardial ischemia-reperfusion injury by inhibiting ferroptosis 46. In contrast, our findings demonstrate that arteannuin B upregulates GDF15 and this induction is essential for ferroptosis in CRC cells. These discrepancies strongly suggest that GDF15's role in ferroptosis is highly context-dependent, varying across tumor types, molecular subtypes, and disease settings.

Mechanistically, our study reveals that GDF15 suppresses the mevalonate pathway by downregulating HMGCS1, which subsequently leads to the reduction of GPX4. In contrast, Huang et al. 45 found that GDF15 knockdown reduces solute carrier family 7 member 11 (SLC7A11) expression, thereby promoting ferroptosis. This suggests that GDF15 may regulate ferroptosis through multiple axes, depending on the cellular context. The opposing roles of GDF15 may also reflect treatment context: our study examines GDF15 induction by arteannuin B, whereas Huang et al. investigated baseline GDF15 suppression. Thus, GDF15 may function as a sensor of metabolic stress, exerting pro- or anti-ferroptosis effects depending on the triggering stimulus.

To our knowledge, this is the first study to show that pharmacological induction of GDF15 by arteannuin B can sensitize CRC cells to ferroptosis, revealing a therapeutic avenue distinct from GDF15's endogenous roles in MSI-H CRC or ccRCC. However, a key limitation of our work is that all mechanistic experiments were conducted in a single CRC cell line (DLD-1). Future studies should validate these results in additional CRC models, including patient-derived organoids or *in vivo* systems representing diverse molecular subtypes, to better define the scope and translational relevance of the GDF15-ferroptosis axis.

In addition, our study demonstrated that arteannuin B regulates ferroptosis via the HMGCS1-mevalonate pathway. TMT proteomics revealed that 48 h treatment with 15 μM arteannuin B markedly reduced the mevalonate-pathway gatekeeper HMGCS1 (Figure 2C), and consequently, subsequent cellular assays confirmed the decrease in its downstream products CoQ10 and cholesterol (Figure 5D-E). In DLD-1^oeHMGCS1^ cells, the downregulation of GPX4 expression induced by arteannuin B was reverted to control levels (Figure 6D-E). In DLD-1^shGDF15^ cells, the elevated lipid peroxidation and MDA levels caused by arteannuin B were substantially reduced (Figure 4E-F), and both HMGCS1 and cholesterol levels were restored (Figure 6A-C). These data suggest that arteannuin B blocks the mevalonate pathway through HMGCS1 rather than the classical target HMGCR, thereby inhibiting GPX4 and triggering ferroptosis. This finding points out that the mevalonate pathway is involved in ferroptosis not only through the HMGCR/GPX4 axis (like statins 47), offering a new molecular target for mevalonate pathway-directed anticancer strategies.

Although our study provides compelling evidence for the anti-cancer mechanism of arteannuin B in colorectal cancer, certain limitations should be acknowledged. Arteannuin B as an ferroptosis inducer is a promising but still in early research. Although our *in vitro* screening encompassed a panel of nine cell lines, the detailed mechanistic dissection was primarily focused on the DLD-1 model, which exhibited the highest sensitivity to the compound. Future investigations involving a broader spectrum of molecular subtypes would further consolidate the universality of the GDF15-mediated pathway. Furthermore, regarding the preclinical models, although the subcutaneous xenograft data strongly support the *in vivo* efficacy, the incorporation of more complex models, such as patient-derived xenografts (PDX) or orthotopic models, would provide a more comprehensive evaluation of its therapeutic potential. Finally, while our preliminary toxicity assessments, demonstrating stable body weight and organ indices, suggest a favorable safety profile, it would be more compelling to include a thorough toxicological analysis, including hematology and histopathology, in future studies to fully pave the way for clinical translation.

## Conclusion

In conclusion, our findings confirm that arteannuin B triggers ferroptosis-like cell death in CRC cells and suppresses xenograft growth. This anti-tumor effect is in association with inhibition of the mevalonate pathway, leading to the disruption of redox homeostasis. Notably, we elucidate that GDF15 contributes to arteannuin B-mediated suppression of HMGCS1 and GPX4, establishing GDF15 as an essential sensitizer for ferroptosis induction. These results provide a mechanistic rationale for targeting the GDF15-mevalonate pathway axis in CRC therapy.

## Supplementary Material

Supplementary figures and tables.

## Figures and Tables

**Table 1:** Organ indices of the nude mice xenografted with DLD-1 cells.

Groups	Organ Indices (mg/g, x̄ ± SD, *n* = 6)			
	Heart	Liver	Spleen	Kidney
Model	5.80 ± 1.55	45.95 ± 1.86	4.28 ± 1.12	13.12 ± 3.43
Blank micelles	4.76 ± 1.62	58.23 ± 2.66	4.68 ± 1.59	14.98 ± 1.18
Arteannuin B micelles	6.06 ± 1.38	58.75 ± 4.81	6.44 ± 1.94	14.54 ± 1.39
Vehicle	5.84 ± 1.50	51.31 ± 2.04	4.76 ± 0.72	14.78 ± 1.46
Arteannuin B	5.98 ± 1.12	52.96 ± 3.74	4.08 ± 1.93	15.40 ± 1.67
5-FU	5.50 ± 1.07	47.99 ± 4.92	5.23 ± 2.05	15.41 ± 1.26

**Table 2:** Organ indices of the nude mice xenografted with DLD-1^WT^ or DLD-1^shGDF15^ cells.

Groups	Organ Indices (mg/g, x̄ ± SD, *n* = 5)			
	Heart	Liver	Spleen	Kidney
DLD-1^WT^/Model	7.52 ± 1.07	53.03 ± 7.45	4.93 ± 0.63	16.18 ± 0.6
DLD-1^WT^/Vehicle	7.53 ± 1.45	52.01 ± 6.58	4.82 ± 0.73	16.01 ± 0.73
DLD-1^WT^/ABMs	7.34 ± 0.53	53.23 ± 9.10	6.07 ± 1.34	16.20 ± 1.95
DLD-1^shGDF15^/ Model	7.90 ± 0.98	56.80 ± 5.02	4.76 ± 0.8	17.56 ± 1.20
DLD-1^shGDF15^/Vehicle	8.10 ± 1.38	54.76 ± 4.58	4.58 ± 0.57	15.78 ± 2.45
DLD-1^shGDF15^/ABMs	7.76 ± 1.48	56.13 ± 4.52	5.81 ± 0.93	17.64 ± 1.09

## Data Availability

The data supporting the findings of this study are available from the corresponding author upon reasonable request.

## Abbreviations

3-MA: 3-methyladenine
5-FU: 5-fluorouracil
7-DHC: 7-dehydrocholesterol
BCA: bicinchoninic acid
ccRCC: clear cell renal cell carcinoma
CoQ10: coenzyme Q10
DCFH-DA: 2,7-dichlorodihydrofluorescein diacetate
DMSO: dimethyl sulfoxide
EGR-1: early growth response protein 1
FDFT1: farnesyl diphosphate farnesyltransferase 1
FDPS: farnesyl diphosphate synthase
Fer-1: ferrostatin-1
GDF15: growth differentiation factor 15
GPX4: glutathione peroxidase 4
HMGCR: 3-hydroxy-3-methylglutaryl-CoA reductase
HMGCS1: 3-hydroxy-3-methylglutaryl-CoA synthase 1
IHC: immunohistochemistry
KEGG: Kyoto Encyclopedia of Genes and Genomes
MDA: malondialdehyde
MVP: mevalonate pathway
Nec-1: necrostatin-1
PPARγ: peroxisome proliferator-activated receptor gamma
ROS: reactive oxygen species
SCAP: sterol regulatory element-binding protein cleavage-activating protein
SDEPs: significantly differentially expressed proteins
SLC7A11: solute carrier family 7 member 11
SREBP2: sterol regulatory element-binding protein 2
TMT: tandem mass tag
Z-VAD-FMK: benzyloxycarbonyl-Val-Ala-Asp(OMe)-fluoromethylketone‌
