# Supplementary material for: Arteannuin B Induces Ferroptosis in Colorectal Cancer Cells via GDF15/HMGCS1/GPX4 Axis
Source: J Cancer. 2026 Jul 20;17(7):1393–407. doi: 10.7150/jca.128611 (PMC13410655; doi:10.7150/jca.128611)
Supplement: Supplementary file 1 — Supplementary figures and tables. [file jcav17p1393s1.pdf]

Supplementary materials

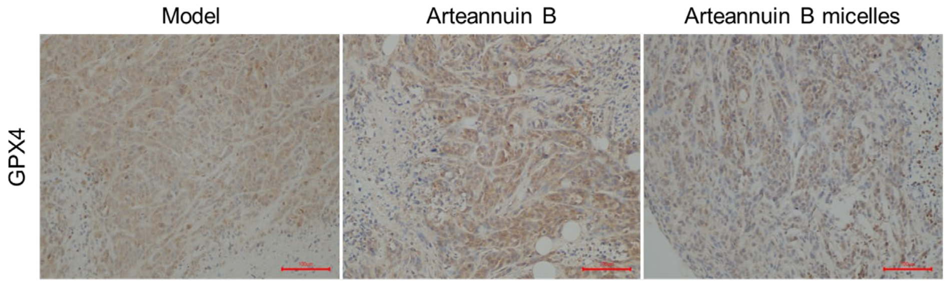

Figure S1. IHC analysis of GPX4 in colorectal cancer-bearing mice (Scale bar = 100 μm).

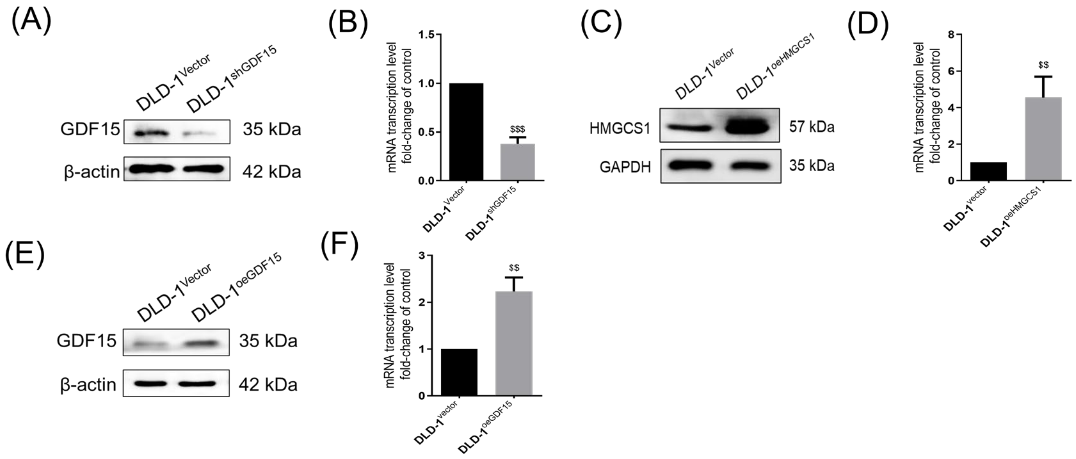

Figure S2. Validation of recombinant cells. (A), (B) Validation of DLD-1^{shGDF15} cells through western blotting and RT-qPCR assays. (C), (D) Validation of DLD-1^{oeHMGCS1} cells through western blotting (C) and RT-qPCR (D) assays. (E), (F) Validation of DLD-1^{oeGDF15} cells through western blotting and RT-qPCR assays. Data are presented as mean ± SD, $n = 3$; $^{\$\$}p < 0.01$, $^{\$\$\$}p < 0.001$ vs. DLD-1^{Vector} cells.

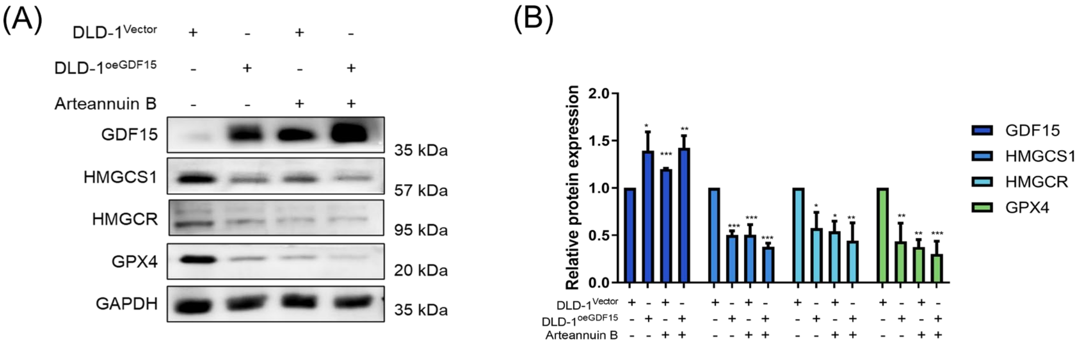

Figure S3. Regulation of multiple proteins by arteannuin B on DLD-1^{Vector} and DLD-1^{oeGDF15} cells. (A), (B) Three independent replicates are shown in western blotting images and quantitative analysis graph. Data are presented as mean ± SD, n = 3; $^{*}p < 0.05$, $^{**}p < 0.01$, $^{***}p < 0.001$ vs. control group.

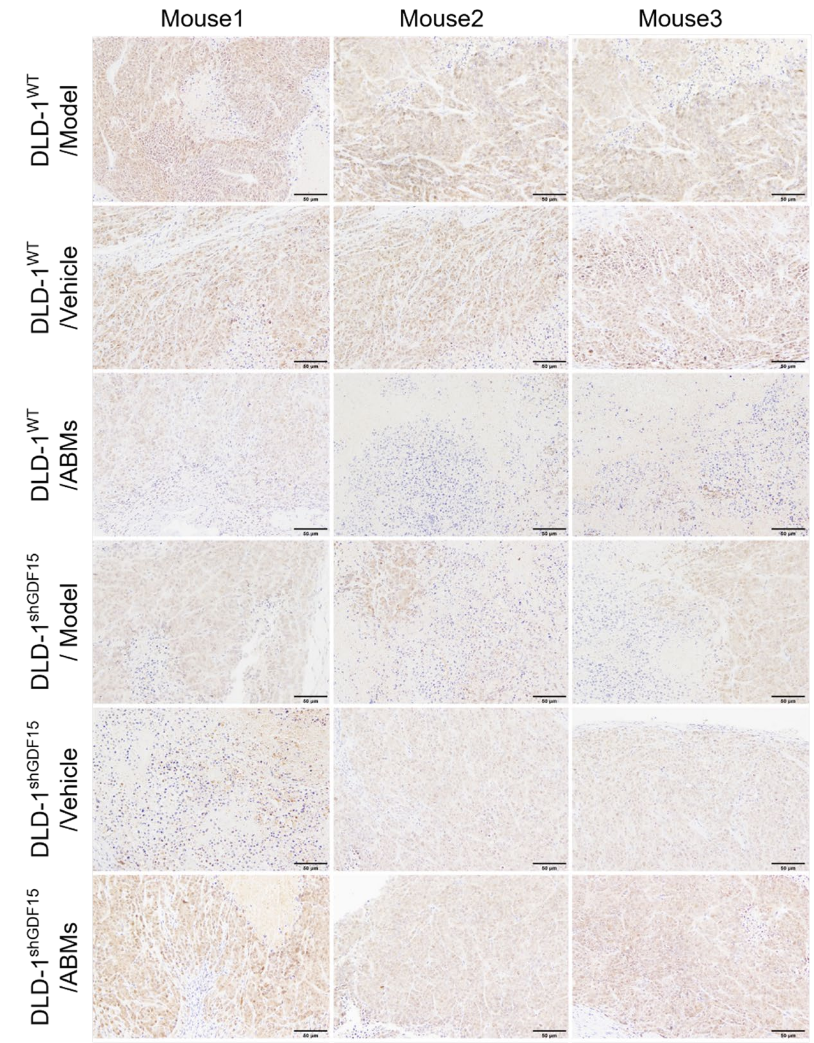

Figure S4. IHC analysis of GPX4 in colorectal cancer-bearing mice (Scale bar = 50 μm).

Table S1. IC_{50} values of arteannuin B on the proliferation of human colorectal cancer cell lines.

Cell Line	IC_{50} Value (μM, $\bar{x} \pm SD$, n = 3)		
	24 h	48 h	72 h
DLD-1	29.97±2.74	7.74±0.53	5.81±0.90
COLO 205	78.54±9.27	45.58±26.54	7.28±4.705
COLO 320	13.58±7.90	8.17±4.96	7.96±6.30
HCT 15	＞100	20.05±4.15	9.38±4.32
HT-29	＞100	22.65±2.58	9.52±4.42
HCT 116	60.17±6.59	15.92±4.28	13.39±3.54
SW480	86.12±7.77	41.12±9.42	31.13±7.48
SW620	＞100	55.75±11.93	40.64±9.63
RKO	＞100	＞100	57.98±28.79
